# Supplementary material for: Drivers of social influence in the Twitter migration to Mastodon
Source: Sci Rep. 2023 Dec 7;13:21626. doi: 10.1038/s41598-023-48200-7 (PMC10703911; doi:10.1038/s41598-023-48200-7)
Supplement: Supplementary file 1 — Supplementary Information. [file 41598_2023_48200_MOESM1_ESM.pdf]

Supplementary Information for “Drivers of social influence in the Twitter migration to Mastodon”

Lucio La Cava[1,*], Luca Maria Aiello[2,3], and Andrea Tagarelli[1]

[1]University of Calabria, Rende, Italy
[2]IT University of Copenhagen, Denmark
[3]Pioneer Centre for AI, Denmark
[*]lucio.lacava@dimes.unical.it

S1.1 Development and signaling of the migration process

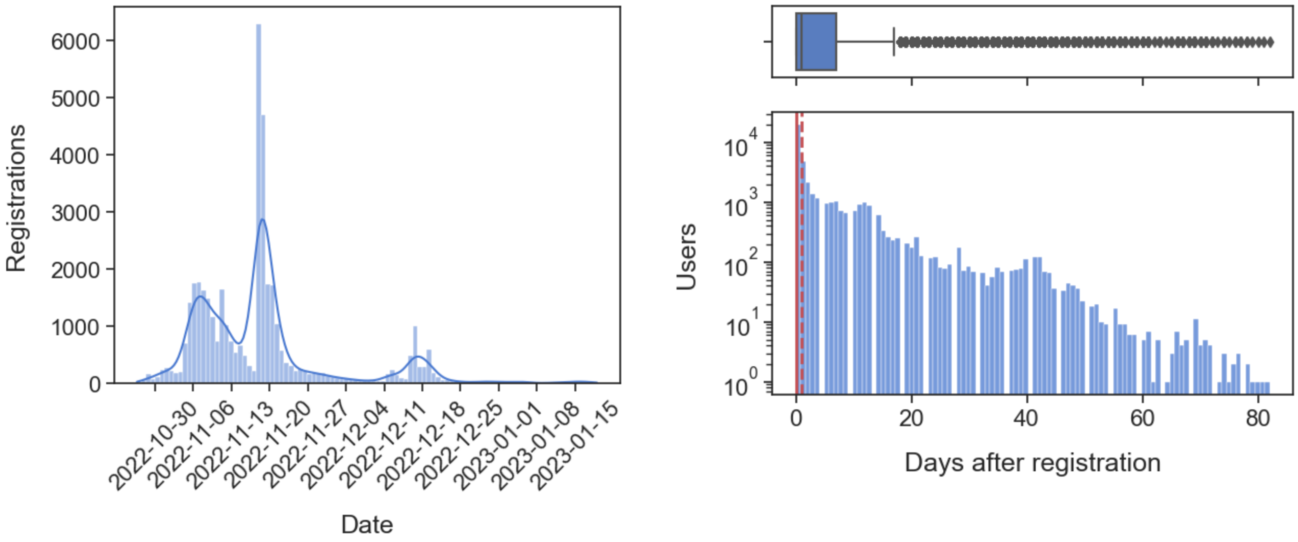

Figure S1. (Left) Registration dates of users migrated to Mastodon between October 26th 2022 and January 19th 2023. (Right) Time delta between migration to Mastodon and signaling of the new Mastodon handles on Twitter; the solid and dashed red lines represent the mode and median of the distribution, respectively.

We analyzed how the #TwitterMigration movement developed as a result of users discussing on Twitter their intention to switch to Mastodon. We hypothesizes that users announcing their Mastodon profiles on Twitter acted as a social trigger that might have persuaded other users to migrate. Given that the date of such announcement is not available for all users, we used the timestamp of Mastodon account creation as a proxy. Figure S1 (left) reports the distribution of registration times after the purchase of Twitter by Elon Musk. Interestingly, some spikes in registrations emerge in response to controversial choices by the new management (e.g., mass layoffs and policy changes). Nonetheless, for our proxy hypothesis to be valid, it is important that the registration time is close to the time in which the Mastodon account was announced on Twitter. We could measure the temporal gap between these two events for a subset of about $41K$ users for whom we have both the registration time on the new platform and the signaling time of the corresponding profile on Twitter. As shown in Figure S1 (right), the time gap is rather short for most users, with mode of 0 days (i.e., less than 24 hours), a median of 1 day, and an average of 5 days.

S1.2 Fitting with the SIR model and with an extended set of users related to the #Twitter-Migration

Fitting the SIR model. In addition to the SIRS model, we experimented with the simpler SIR model. We observed that the SIR model could replicate the observed trend of the cumulative number of migrated users equally (same R_0 and MAPE values) with the SIRS model, as reported in Figure S2 (left). However, in this regard we point out that although this may seemingly suggest that from a macroscopic point of view both models perform identically, this turns out to be a borderline case, as in

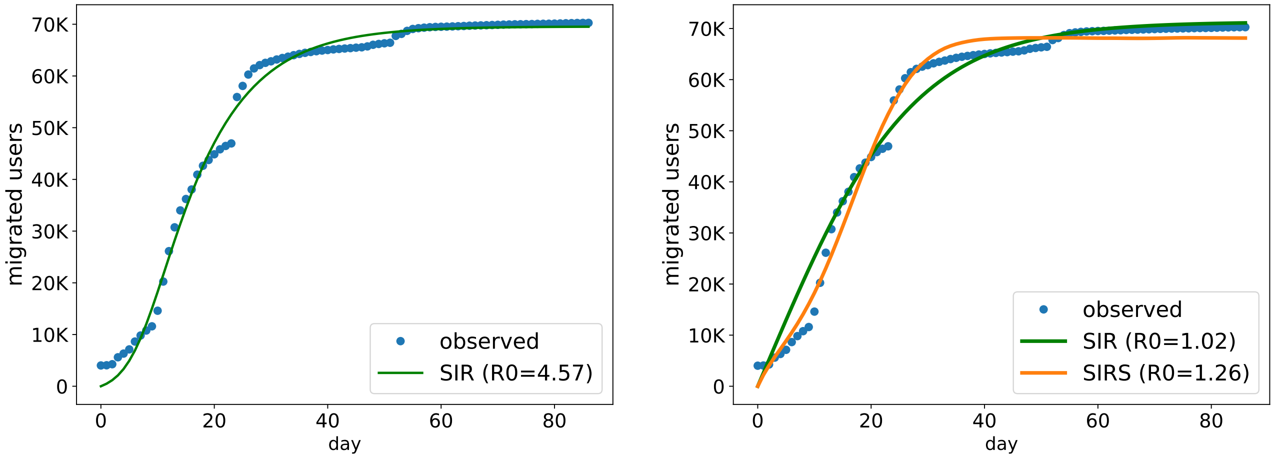

Figure S2. Cumulative number of Twitter users migrated to Mastodon over the course of 3 months since Elon Musk's acquisition of Twitter. (Left) Fit estimated with the SIR compartmental epidemiological model. (Right) Fit estimated with the SIR and SIRS compartmental model with the set of users corresponding to the population including all users who have discussed Mastodon on Twitter.

general the SIRS model has been shown to be superior in terms of goodness of fit (cf. Results) due to its ability to model reiterated commitment in our infectiousness scenario.

Expanding the population. Our primary focus in this work is on the analysis of the main drivers of social influence behind the #TwitterMigration movement, and accordingly we considered as the population underlying our epidemic models the set of users eventually migrated to Mastodon. Nonetheless, to conduct a comprehensive study, we extended our evaluation by broadening the set of users representing our population. Specifically, this expanded set includes $\sim$ 540K users who have engaged in discussions involving Mastodon between October 26th 2022 and January 19th 2023, regardless of their final decision to migrate or not. Although this choice provides an underestimation of the actual process due to the inclusion of potential noise in the population, our SIR and SIRS models estimated an R_0 value of 1.02 and 1.26 (see Figure S2, right), respectively. Since R_0 is greater than 1, this confirms growth of the infection process underlying social influence.

S1.3 Further details on communities

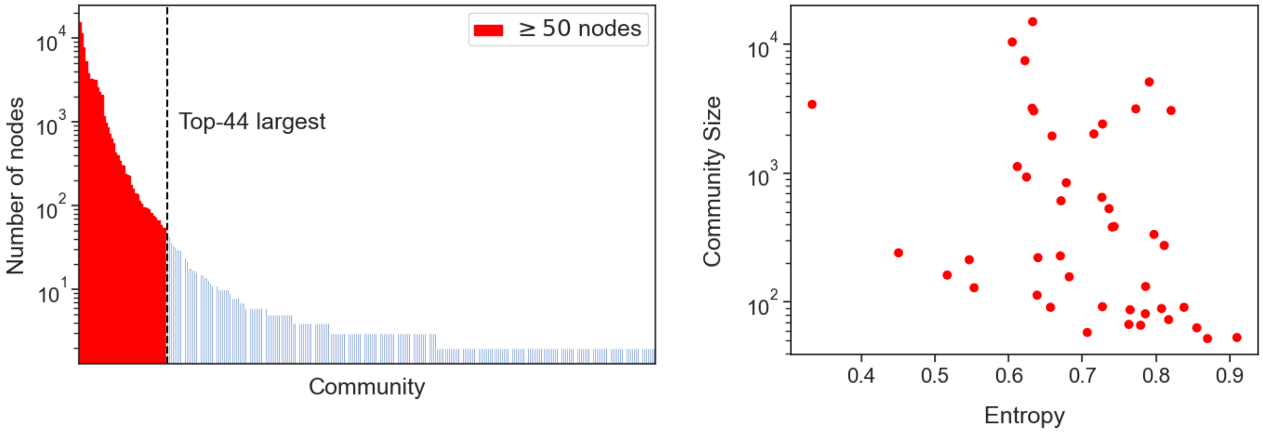

Figure S3. (Left) Distribution of users across the communities found via the Louvain method; red-colored communities contain at least 50 users. (Right) Scatterplot showing the relationship between community topical entropy and size for the top-44 largest communities.

To investigate the spreading of the #TwitterMigration's infectiousness across communities, we focused our analysis on a specific group of nodes containing a sufficiently representative number of users. Specifically, by referring to the distribution

reported in Figure S3 (left), it is possible to observe that most users turn out to be clustered in a few communities, with a long tail of communities containing a few members, or even singleton nodes. Consequently, for our analysis, we selected the set of communities having at least 50 users, which gives us coverage of 98% of the set of migrated users, corresponding to the top-44 largest communities. In this regard, we also complement our findings on the relationship between community size and topical entropy by resorting to Figure S3 (right), which shows the (moderate) negative correlation we spotted between these two quantities across the top-44 largest communities.

S1.4 Fitting compartmental models with the largest communities

Figure S4 shows how the SIR and SIRS model fit the migration data for the top-15 largest communities by number of users. In line with our findings (cf. Results), both the SIRS and SIR models are able to accurately shape the migration process, albeit with different R_0 (with mean values of 5.08 for SIRS and 3.92 for SIR for the top-44 largest communities).

S1.5 Experimenting with regression models to predict community-specific R_0

Table S1 reports the regression model fittings for predicting community-specific R_0 based on the full set of topological and social features we leveraged in our analyses. Table S2 reports the fitting of a Least Absolute Shrinkage and Selection Operator (LASSO) regression model on the combination of the best-performing features for predicting R_0 (cf. Results).

**Predicting R_0 from:**				**Predicting R_0 from:**			
**Topological Features**				**Social Features**			
**Feature**	β	**SE**	p	**Feature**	β	**SE**	p
Density	-0.055	0.967	0.955	Support	0.040	0.188	0.834
Reciprocity	-0.123	0.235	0.604	Knowledge	0.507	0.161	0.003
Assortativity	-0.015	0.270	0.955	Conflict	-0.043	0.171	0.802
Std. Deg. Centr.	0.230	1.345	0.865	Power	0.248	0.157	0.124
Transitivity	-0.426	0.604	0.485	Similarity	-0.103	0.220	0.641
Avg. Clust. Coeff.	-0.175	0.500	0.729	Status	-0.126	0.194	0.519
				Trust	0.098	0.153	0.526
				Identity	0.511	0.141	0.001
Durbin-Watson stat. = 2.661		R^2_{adj} = **0.140**		Durbin-Watson stat. = 2.038		R^2_{adj} = **0.395**	

Table S1. Ordinary Least Squares regression model fittings for the prediction of R_0 from the full set of topological (left) and social (right) features. β coefficients describe the contribution of each feature to the outcome, along with the standard errors (SE) and statistical significance (p-values). Auto-correlation is evaluated via the Durbin-Watson statistic (values closest to 2 indicate no auto-correlation). Regression results are reported via adjusted R^2.

**Predicting R_0 from:**	
**Topological & Social & Activity Features**	
**Feature**	β
Density	-0.274
Knowledge	0.244
Identity	0.246
Commitment	0.240
R^2_{adj} = **0.525**	

Table S2. Least Absolute Shrinkage and Selection Operator regression model fitting for the prediction of R_0 from a combination of topological, activity, and social features. β coefficients describe the contribution of each feature to the outcome. Penalty term is set to 0.1. Regression results are reported via adjusted R^2.

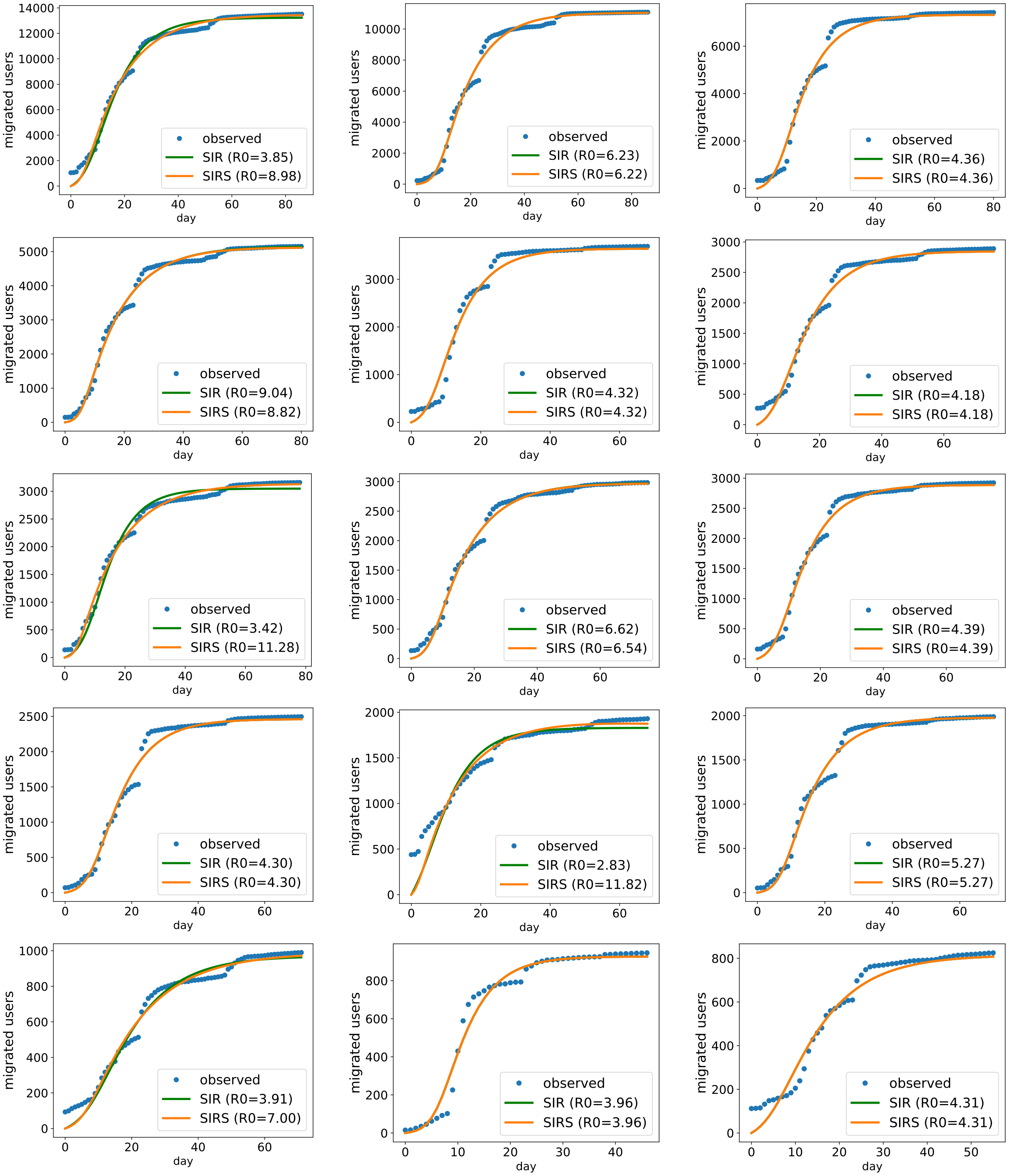

Figure S4. Fitting with the SIR and SIRS compartmental epidemiological models and associated R_0 values for the top-15 largest communities by Louvain, reported in descending order row-wise, of the cumulative number of Twitter users migrated to Mastodon over the course of 3 months since Elon Musk's acquisition of Twitter.

S1.6 User activity in the months following the migration

To assess the effectiveness of the migration from Twitter to Mastodon almost a year after the Musk's buyout, we examined the activity levels of migrated users. To this aim, we utilized Mastodon's official APIs to retrieve the date of the most recent

Check	Perc.
Account existence	95.13%
Active after 1 month	69.26%
Active after 3 months	54.60%
Active after 6 months	44.73%
Active in the last 3 months	37.61%
Active in the last 2 months	32.78%
Active in the last month	27.50%
Volume $\geq$ Q3 freshness	73.56%
Volume $\geq$ Q2 freshness	90.13 %

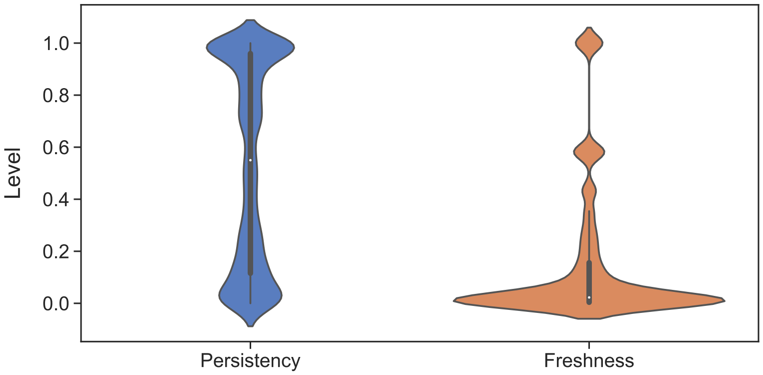

Figure S5. Percentage of accounts still existing and active w.r.t. different temporal checkpoints. (Right) Violin plots for the persistency and freshness levels of migrated users.

post made by migrated users. This check was performed in mid-October 2023. The presence of a valid payload not only confirmed the user's ongoing activity (i.e., post creation) on Mastodon, yet also allowed us to determine its extent. Figure S5 (left) summarizes the key findings from our analysis. Remarkably, more than 95% of the migrated users maintained their account on Mastodon. Furthermore, while we observed a decline in activity due to the initial surge following the collective migration, we found that almost half of the migrated users remained active six months after the migration, suggesting interest in well-rooted settlement from a considerable fraction of migrated users. We took a closer look at users who made particular efforts to remain active even after a substantial period following the surge in migrations. To this aim, we calculated the fraction of users who continued to contribute by creating content in the last few months. Notably, as reported in Figure S5 (left), more than a quarter of migrated users were observed to be actively posting during the month leading up to our mid-October 2023 check. These users constitute a resilient segment of the migrated population that was able to endure the initial noisy growth due to the curiosity to try out a new platform, thus effectively managing to cave out a new social space and maintaining their engagement and participation in the community. We further delved into such an investigation by computing, for each migrated user i, the corresponding *persistency* and *freshness* levels. The former is defined as $p_l(i) = t_p(i)/t_m(i)$ and indicates the duration of which the user i has been active (considering the creation of posts) on the platform $t_p(i)$, compared to the time has passed since migrating $t_m(i)$. The latter has been defined in previous work[1] as $f_l(i) = 1/\log_2(2+t_\Delta)$, where t_Δ indicates the number of days elapsed between the date of checking the user's activity (i.e., mid-October 2023) and the date of the last post created by the user. These two complementary scores were normalized in $[0,1]$ and serve as proxies for the migrated users' degree of persistency and propensity to contribute, respectively. As reported in Figure S5 (right), migrated users almost split in a bipartite fashion. Indeed, while we observed a moderate fraction of migrated users not actively engaging in the new platform, most of them were found to be clustered at high persistency levels, with observable peaks around the maximum value. Similarly, despite observing that a large fraction of migrated users are not particularly keen to contribute, we found two noticeable groups exhibiting mid and high values of freshness, respectively, thus actively contributing with fresh content on the new platform. We further evaluated the extent of this propensity to contribute, unveiling very intriguing traits. Indeed, as reported in Figure S5 (left), these "fresher" users were responsible for generating nearly the entire volume (90.13%) of posts by migrated users on the new platform. This is particularly evident among those in the last quartile of freshness value (73.56%), which hence act as "super-users". These intriguing findings underscore the pivotal role played by these super-users in sustaining the post-migration ecosystem and pave the way for further investigations. Finally, we explored the potential connection between the level of persistency/freshness and the underlying social influence in the migration process. Remarkably, we observed a non-negligible correlation between the R_0 value of the SIRS model and the corresponding persistency ($p = 0.491$) and freshness ($p = 0.394$) levels of the top-44 communities on which we narrowed our focus within our study. This finding, coupled with the replication of more than 40% of the social ties after the migration (cf. Results), poses a stepping point for the proper understanding of the growing migratory phenomena between social platforms, thereby warranting further investigations.

References

1. Tagarelli, A. & Interdonato, R. Time-aware analysis and ranking of lurkers in social networks. Soc. Netw. Analysis Min. **5**, 1–23 (2015).
